# Microbe Profile: *Wolbachia*: a sex selector, a viral protector and a target to treat filarial nematodes

**DOI:** 10.1099/mic.0.000724

**Published:** 2018-10-12

**Authors:** Mark J. Taylor, Seth R. Bordenstein, Barton Slatko

**Affiliations:** ^1^​ Liverpool School of Tropical Medicine, Pembroke Place, Liverpool, L3 5QA, UK; ^2^​ Department of Biological Sciences, Vanderbilt University, Nashville, TN, USA; ^3^​ Department of Pathology, Microbiology, and Immunology, Vanderbilt University, Nashville, TN, USA; ^4^​ Vanderbilt Institute for Infection, Immunology and Inflammation, Vanderbilt University, Nashville, TN, USA; ^5^​ Vanderbilt Genetics Institute, Vanderbilt University, Nashville, TN, USA; ^6^​ New England Biolabs, Inc., 240 County Road, Ipswich, MA 01938, USA

**Keywords:** *Wolbachia*

## Abstract

*Wolbachia* is the most widespread genus of endosymbiotic bacteria in the animal world, infecting a diverse range of arthropods and nematodes. A broad spectrum of associations from parasitism to mutualism occur, with a tendency to drive reproductive manipulation or influence host fecundity to spread infection through host populations. These varied effects of *Wolbachia* are exploited for public health benefits. Notably, the protection of insect hosts from viruses is being tested as a potential control strategy for human arboviruses, and the mutualistic relationship with filarial nematodes makes *Wolbachia* a target for antibiotic therapy of human and veterinary nematode diseases.

## Taxonomy

Domain *Bacteria*, phylum *Proteobacteria*, class *Alphaproteobacteria*, order *Rickettsiales*, family *Anaplasmataceae*, tribe *Wolbachieae*, genus *Wolbachia*, species *Wolbachia pipientis.* The type species was first described in the mosquito *Culex pipiens* in 1924, but *Wolbachia* are most commonly referred to by only their genus. Strains are labelled according to their host and supergroup in shared hosts, e.g. *w*AlbB, from *Aedes albopictus* supergroup B.

## Properties

*Wolbachia* are obligate endosymbiotic α-proteobacteria that are most closely related to the genera *Ehrlichia* and *Anaplasma*. They are pleiomorphic, ranging from 0.2 to 4 µm in size, and reside in an obligate intracellular niche within host-derived vacuoles. The cell is Gram-negative, with an inner and outer membrane, but has lost much of the typical cell wall structure, including peptidoglycan. Extracellular transfer occurs briefly in nematodes, but is restricted to the transfer from hypodermis to ovary within the same host. In arthropods transfer between different hosts occurs frequently and even plants can act as a temporary bridging ‘host’. Because *Wolbachia* are primarily transmitted vertically through the host maternal germline, they possess an extraordinary ability to alter core host cellular and developmental processes to favour infected females. This includes altering host chromosome condensation and cell cycle timing, sex determination pathways, apoptosis, stem cell biology and axis determination during early embryogenesis.

## Genome

Over three dozen *Wolbachia* genomes from arthropods and nematodes have been fully or partially sequenced, as referenced in GenBank. Not all genomes are complete in a single contig, mostly due to the presence of repetitive DNA, which makes final assembly a challenge. Nevertheless, for most of these genomes, annotations of the gene sets exist. There are also many more additional nucleotide sequences from other *Wolbachia* genomes in databases, such as for the *wsp* gene that encodes an outer membrane protein. Beginning with the sequencing of the first *Wolbachia* genomes of *w*Mel from *Drosophila melanogaster* and *w*Bm from *Brugia malayi,* the goals were to (1) understand the unique biology of *Wolbachia–*host reproductive manipulations (arthropods), (2) identify potential drug targets (filarial nematodes), (3) delineate the nature of host–symbiont mechanisms (parasitic or mutualist) and (4) detail the phylogenetic/evolutionary relationships of *Wolbachia*. Within these objectives, a number of gene/gene systems have been under investigation to draw parallels among the diverse systems (e.g. heme biosynthesis, DNA metabolism, type IV secretion system, prophage WO sequences and lateral gene transfers). *Wolbachia* genomes are generally in the 1 Mb range, but can be as large as 1.8 Mb (*Folsomia candida*) or as small as 0.9 Mb (*Dirofilaria immitis*). Generally, *Wolbachia* genomes from filarial nematodes are smaller than their arthropod counterparts, largely due to the presence of WO phage transfers in the latter. In general, the gene ‘counts’ range from 800 to 1000. In numerous cases, *Wolbachia* DNA has been shown to have been acquired by host chromosomes (lateral gene transfer) and some of these sequences appear to be functional. In several cases, virtually the entirety of the *Wolbachia* genome has been transferred to the host (*Drosophila ananassae* and *Callosobruchus chinensis). Wolbachia* are not simply passive travellers.

## Phylogeny

The genus *Wolbachia* contains 16 divergent lineages that are identifiable in the current ‘supergroup’ classification system. They are denoted A–Q, although supergroup G was decommissioned due to it being a recombinant. Most, but not all, supergroups show host range restriction to either arthropods or nematodes, and several arthropod-associated lineages show extensive host switches between different arthropod species. Accumulating discoveries of new supergroups suggest there will be continued detection of additional supergroups, especially in undersampled host taxa. There are two major challenges confronting *Wolbachia* phylogenetics. First, the long-term stability of a phylogenetic framework is dependent on genomic data that do not currently exist for many *Wolbachia* supergroups. Second, the genetic divergence between *Wolbachia* and its sister genera causes a problematic long-branch attraction artifact that currently precludes resolution of the root of the *Wolbachia* tree and therefore the direction of evolutionary and ecological changes in genome size, host range and symbiotic states across the genus.

## Key features and discoveries

*Wolbachia* are one of the great pandemics of life from a biodiversity perspective because they are estimated to occur in millions of invertebrate species, including 40 % of all arthropod species. They evolved an arsenal of host reproductive manipulations that propagated their worldwide prevalence in arthropods, including feminization, parthenogenesis, male-killing, meiotic drive and cytoplasmic incompatibility [1]. These phenotypes serve to selfishly increase the frequency of infected females in a host population by enhancing the fitness of infected females, the transmitting host. While each of these modifications is well characterized at a phenotypic level, one of the pre-eminent questions in the field is how does *Wolbachia* hijack animal reproduction to facilitate its global spread and incidence? Recently, a set of multi-omic, transgenic and cytological approaches uncovered the *cifA* and *cifB* genes responsible for the most common adaptation, cytoplasmic incompatibility, in the eukaryotic association module of prophage WO in the *w*Mel *Wolbachia* genome [2] and others. *cifA* and *cifB* induce a sperm modification that is lethal to uninfected embryos, unless the same *cifA* product is present in infected embryos to rescue the lethality.

An exciting development from studying *Wolbachia* in arthropods is the ability of *Wolbachia* to protect their hosts from pathogens, in particular RNA viruses, and the ability of cytoplasmic incompatibility to rapidly drive *Wolbachia* into naïve populations. *Aedes aegypti*, the insect vector of arboviruses (dengue, chikungunya, Zika and yellow fever) is not naturally infected with *Wolbachia*, yet the bacterium has been successfully introduced into wild populations [3]. The World Mosquito Program (https://www.worldmosquitoprogram.org) and MosquitoMate (https://mosquitomate.com) are currently field testing the large-scale release of *Wolbachia*-infected *Aedes* in communities prone to arboviral outbreaks and randomized cluster trials have been launched to provide evidence of the effectiveness of this approach.

*Wolbachia* are also present in most human filarial nematodes, where they have taken an alternative evolutionary trajectory as strict mutualists. Whilst they benefit the nematode host, their release into the human or animal host drives inflammatory responses associated with disease pathogenesis [4]. *Wolbachia* are essential for worm development, fertility and longevity, and as such have proved to be an ideal target for drug therapy [5]. Antibiotic treatment leads to a radical cure of patients suffering with onchocerciasis or lymphatic filariasis and the international Anti-*Wolbachia* Consortium (A-WOL, http://awol.lstmed.ac.uk) is developing new drugs to provide improved treatments for human filariasis to ensure that the long-term goal of global elimination of this public health problem is achieved.

The exponential expansion of *Wolbachia* knowledge shows no sign of diminishing, with exciting new areas surrounding their role in influencing host behaviour, microbiome composition and manipulation of core host cellular and development processes [6]. Future studies will no doubt unravel additional biological consequences of *Wolbachia* infection and insights into the evolutionary origins of endosymbiosis.

## Major questions

- What is the complete genetic and mechanistic basis of reproductive parasitism in arthropods?
- What *Wolbachia* mechanism renders host arthropods refractory to arboviruses?
- What processes and provisions serve the mutualism in nematodes?
- Which interdomain lateral gene transfers between *Wolbachia* and hosts are functional, and which are fossils?
- Which *Wolbachia* lineage arose first, and what is the origin and functional legacy of bacteriophage WO in the genus?
